# Novel Time-Series Forecasting Method to Enhance Accuracy of Real-Time EEG Detection for BCI-Based Neurofeedback Motor Training in Individuals with Cerebral Palsy and Other Neurological Disorders

**DOI:** 10.3390/bioengineering13050561

**Published:** 2026-05-16

**Authors:** Andrew Gravunder, Amanda Studnicki, Julia Kline, Ahad Behboodi, Thomas C. Bulea, Diane L. Damiano

**Affiliations:** 1 Neurorehabilitation and Biomechanics Research Section, Rehabilitation Medicine Department, Clinical Center, National Institutes of Health, Bethesda, MD 20892, USA; andrew.gravunder@nih.gov (A.G.); amandastudnicki@gmail.com (A.S.); jekline2@gmail.com (J.K.); abehboodi@unomaha.edu (A.B.); thomas.bulea@nih.gov (T.C.B.); 2 Department of Biomechanics, University of Nebraska Omaha, Omaha, NE 68182, USA

**Keywords:** electroencephalography, neurorehabilitation, ankle dorsiflexion, moving averages, motor function, recovery

## Abstract

Real-time detection of motor intent using electroencephalography (EEG) with high accuracy remains a technical challenge for neurorehabilitation. Brain–computer interface-based neurofeedback training (BCI-NFT) paradigms need to detect pre-movement EEG to activate robotics or electrical stimulation nearly simultaneously with movement to promote neuroplasticity. We present a novel detection method commonly used in time-series forecasting (e.g., stock market trends), identifying crosses in fast (short) and slow (long) moving average windows to identify negative deflections in slow movement-related cortical potentials (MRCPs) or event-related desynchronization (ERD) within −400–+100 ms of movement onset. We recorded EEG data from the Cz electrode during our cued ankle dorsiflexion BCI-NFT paradigm in four adult participants, two neurotypical and two with cerebral palsy. Simulated real-time offline analyses demonstrated an 85.9% mean true positive rate and 14.1% false positive rate of detecting motor intent at a mean −182 ms from movement onset. We further evaluated whether the detection indicated a MRCP and/or ERD, with MRCP detected in 70–80% of trials in three participants, but high ERD detection (87%) instead in the other. Preliminary results indicate that this approach offers a straightforward, accurate, and well-timed method for real-time EEG detection during neurofeedback training and as a control signal for brain–computer interfaces.

## 1. Introduction

Individuals with cerebral palsy (CP) and chronic stroke often experience selective motor control impairments (e.g., inability to open their hand to grasp objects or dorsiflex their ankle to avoid tripping) that may not improve significantly with task-specific training alone. Neuromodulation techniques such as transcranial magnetic stimulation (TMS), transcutaneous direct current stimulation (tDCS), and, more recently, epidural spinal stimulation have been combined with motor training to augment rehabilitation outcomes [1,2,3,4]. While these paradigms demonstrate largely positive effects, utilizing one’s own brain signals, detected by electroencephalography (EEG), to augment movement during training through Brain–Computer Interface Neurofeedback Training (BCI-NFT) may offer a more ecologically valid and efficient approach to promote adaptive neuroplasticity and improve motor function [5].

EEG has been utilized in medicine for decades, primarily to identify and localize seizure activity, and has evolved considerably over time to enhance diagnosis and treatment of psychiatric disorders such as depression, sleep, and attention deficit-hyperactivity disorder, among others [6]. In this paper, we are focusing on its more recent use in functional neuroimaging and neurorehabilitation of neuromotor disorders. From a historical perspective, EEG is the oldest neuroimaging technology, with the first recording of an electroencephalogram by Hans Berger in 1924 during a neurosurgical procedure on a 17-year-old boy [7,8]. He later pioneered non-invasive scalp recordings during movement and was the first to describe alpha and beta waves and how these change during different brain states. EEG records electrical discharge from large groups of activated pyramidal neurons from the scalp, with high temporal but low spatial resolution due to volume conduction and contamination with EMG activity from nearby muscles, and other non-cortical signals that need to be removed during processing. The precise temporal resolution of EEG makes it particularly useful for brain–computer interface or neurofeedback applications and for studying brain activation during specific movement or task events or phases, made possible through advances in EEG processing and analysis techniques [9]. Unlike magnetic resonance imaging, EEG also offers the advantage of being less affected by excessive head movements, common in many neurological disorders, or an inability to remain still for prolonged periods in younger or less cooperative participants, thus expanding the ability to detect brain activity across a far wider range of experimental tasks and patient populations.

Promising neurorehabilitation outcomes from BCI-NFT have been primarily demonstrated in individuals post-stroke, with methods that monitor brain signals associated with movement intention or execution using invasive or, in most cases, surface EEG, and then utilizing these to activate robotic or electrical stimulation devices to augment motor training [10]. Closer timing between the desired and/or elicited motor response and the detection of the intent to produce that response is highly advantageous for improving user experience and performance. As a result, many BCI applications focus on slow (0.5–5 Hz range) movement-related cortical potentials (MRCPs), which show a decrease in electroencephalography (EEG) amplitude over sensorimotor channels starting around 2 s before movement onset, often thought to reflect movement planning and preparation [11].

The negative electrical potential that consistently precedes self-paced movements is termed the Bereitschaftspotential (BP), first identified more than 60 years ago [12]. It has also been called the readiness potential, or, alternately, the Contingent Negative Variation (CNV) when it occurs during cued real or imagined movement [13]. A gradual negative deflection in this slow frequency range begins approximately 1–2 s prior to movement onset, becomes steeper around 400–500 ms prior to movement, and then reverses direction around the time of movement onset [11]. The two pre-movement subcomponents have different cortical origins: the early deflection starts in the bilateral presupplementary and supplementary motor areas and shortly thereafter in the premotor regions, and the later component is most apparent contralateral to the movement side, likely in the primary motor cortex [14,15,16]. For BCI-NFT applications, detection of this later pre-movement EEG signal could be used to activate either a functional electrical stimulation (FES) or a robotic device for motor assistance and sensory augmentation as close to movement onset as possible [17].

According to the Hebbian principle that “neurons that fire together, wire together” [18,19,20], the timing of stimulation for neuromodulation is crucial in neurorehabilitation for restoring an impaired connection between efferent motor commands and afferent sensory feedback to promote functional recovery for patients with stroke [21], spinal cord injury [22], and CP [10,23]. Mrachacz-Kersting and colleagues found that stronger long-term potentiation, measured via motor-evoked potentials with TMS, occurred when peripheral stimulation was precisely timed with maximal motor-related cortical activity [24]. The consensus from an expert group meeting proposed that a maximal latency, between detection and activating sensory feedback, of 200–300 ms was likely optimal for inducing Hebbian plasticity, but also agreed that this was an open question for the field that may differ across patient groups [25]. If CNV detection is successful just prior to movement, even with brief delays in both neural transmission and device activation, convergence can occur very close to movement onset. A mismatch in timing caused by inaccurate real-time MRCP detection or from alternate strategies that use electromyography or kinematic markers reflecting movement execution which may be too late could potentially disrupt rather than promote neuroplasticity. Several authors have also proposed that event-related desynchronization (ERD) in the mu and beta bands, which can occur up to 2 s prior to movement onset, may also be effective for real-time detection of movements [26,27]; however, Planelles and colleagues [23] concluded that accuracy of ERD detection, even when optimized, showed a true positive rate (TPR) of about 70%, and a false positive rate (FPR) of about 28%, indicating that a device may activate motor assistance even when the person was not choosing to move. They noted that concurrent methods utilizing slow MRCPs and ERDs tended to report higher TPR and lower FPR (e.g., Niazi et al., 2011) [28], both of which are more desirable for associative learning BCI and rehabilitation applications.

Highly accurate real-time detection of pre-movement EEG signals related to the desired motor task remains a major challenge in neurorehabilitation. Although several studies purport to utilize real-time detection of pre-movement MRCPs in neurofeedback paradigms, the literature review of the various techniques used to maximize detection accuracy did not reveal a consistently superior and/or replicable methodology. The main factors limiting real-time detection of MRCPs include: (1) decreased accuracy due to the low signal-to-noise ratio (SNR) inherent in EEG signals that is magnified in single trial ‘online’ analysis, as opposed to post hoc offline analysis where multiple epochs can be averaged, and (2) increased latency due to associated computational time of the method chosen to process the signal. Because MRCPs are low-frequency low-amplitude cortical potentials, noise and various artifacts can easily overpower them [23,29]; thus, reliably extracting amplitude and timing-based features from single trials remains a challenge, even with sophisticated filtering and artifact-removal techniques. In many clinical populations with motor deficits, MRCP amplitudes may be even more suppressed than in young healthy controls [29].

Despite numerous signal processing innovations, no one method has yet demonstrated superiority, with most published MRCP-based detection algorithms limited by precision, real-time feasibility, or transparency in performance reporting. Techniques such as constrained independent component analysis (cICA), Laplacian filtering, and template matching have demonstrated moderate improvements in signal-to-noise ratio (SNR) and detection latency in controlled settings. For instance, cICA was shown to achieve up to 87% true positive rate with minimal latency (~30 ms) in an offline study of ankle dorsiflexion with healthy subjects [30], and optimized Laplacian filters have enabled pre-movement detection in stroke survivors of up to 200 ms before movement onset [29]. Matched filtering is a form of template-based detection in which a canonical MRCP waveform is convolved with actual EEG signals to identify movement intention. While effective under controlled conditions, it is sensitive to inter- and intra-subject variability. Niazi and colleagues proposed an optimal spatial filter to improve robustness, achieving a reported 82.5% accuracy offline in healthy individuals [28]. Template-based methods, including matched filters and cICA, as well as common spatial patterns (CSP), rely on consistent MRCP morphology, are sensitive to noise and trial-to-trial variation, and typically require individualized calibration. Classifier-based approaches, which require a set of data to train algorithms for detecting the MRCP presence before movement initiation, have also been deployed, with accuracies approaching 80% in the detection of lower extremity movements [27,31]. More recent approaches, such as non-linear spatiotemporal filters [32] and deep learning-based classifiers [33], show promise, but still face challenges when applied in real-world and/or asynchronous settings [11].

Yet another challenge is that, although MRCP-based systems for motor training that trigger external devices such as FES or robotic assistance have demonstrated clinical effectiveness in research settings [34,35], their translation into routine rehabilitation practice remains limited. Despite the breadth of classifier types, including Support Vector Machines (SVMs), as demonstrated by Bhagat et al. [36] with 79 ± 18% classification accuracy and a 23 ± 20% false positive rate, these and other approaches remain constrained to offline environments and are not well suited for clinical use. In a 2018 study, Mrachacz-Kersting et al. [35] decided to forgo real-time detection and instead implemented a feedforward design based on first detecting the average timing of MRCP peak negativity relative to movement onset and then using this timing information to deliver what they referred to as “brain state-dependent stimulation.” Their choice of this approach underscores the field’s ongoing technical limitations and, that, despite encouraging developments, to the best of our knowledge, no MRCP-based BCI to date fully meets the combined requirements of a high degree of accuracy, real-time responsiveness, and clinical feasibility.

Over the past several years, our group has been developing a BCI-NFT system using slow MRCP signals from surface EEG to activate electrical stimulation assistance during active motor training of ankle dorsiflexion in children with CP. We implemented multiple strategies in our efforts to develop highly accurate real-time detection, including the use of transfer learning, which attempted to identify the peak in the negative deflection prior to movement time in a cued paradigm [37]. Since our paradigms involve active motor training where most participants are able to voluntarily activate the tibialis anterior muscle to some extent, we first implemented a hybrid approach based partly on that of Mrachez-Kersting [35], that was partly, but not exclusively, real time. In this method, a participant would be stimulated if peak negativity was detected prior to movement onset, or alternatively near the conclusion of the 500 ms pre-movement window, if real-time detection of the negative potential had not yet occurred. We have also piloted several other strategies advocated in the literature (e.g., cICA, template matching) but none demonstrated improved real-time detection accuracy within our paradigm. We then observed that raw EEG data prior to movement, in comparison to a resting phase, appeared to resemble similar appearing trends to time-series datasets used to predict weather, stocks, or sales trends.

Therefore, to address the ongoing challenge in the field of highly accurate and precisely timed real-time EEG detection for BCI applications, and specifically here for BCI-NFT, we propose that identifying features in the EEG signals, i.e., a clear downward trend followed by a sharp upward deflection in the slow MRCP, also termed the CNV, or ERD in the alpha (8–13 Hz) and low beta frequency bands (13–20 Hz), that becomes most pronounced just prior to movement, may be detectable by using a novel time-series forecasting approach. Our algorithm involves the use of moving averages (MA) which have been previously applied as filters to smooth or reduce high-frequency fluctuations, including noise, from EEG signals given their straightforward implementation and effectiveness. However, moving averages are also used to highlight long-term trends in time-series data like trend prediction, or most commonly, in forecasting financial time series [38]. Here, we opted to utilize a Fast shorter-term MA and a Slow longer-term MA over a specific pre-movement time window to identify a cross-over point which occurs when the Fast MA crosses under or over the Slow MA [39]. The Fast MA crossing under the Slow MA indicates the start of a downward trend, or the local maximum. This may reflect either a steep negative decline, e.g., CNV or the onset of ERD. Alternatively, an opposite pattern of the Fast MA crossing over the Slow MA would indicate a previous local minimum and an upward trend or, perhaps in this case, a sharp upward deflection after a steep negative decline in the EEG signal close to movement onset. When this occurs in a time-series prediction, the probability would point toward an upward trend and a previous local minimum.

We present here preliminary results from the evaluation of this novel real-time EEG detection strategy, which, to our knowledge, has not been previously utilized in BCI applications, including those for motor substitution as well as for motor training. We utilized our existing BCI-NFT paradigm to collect multiple sets of data in four participants, two of whom had cerebral palsy (CP) and two of whom had no neurological or other disorders, during a simulated training session that required participants to perform ankle dorsiflexion when cued. The only difference to participants from an actual training session was that no electrical stimulation was provided to them when detection occurred, although it was connected to the system for data collection purposes. Once the dataset was obtained, performance analysis and method refinement were completed offline, with data fed into the detection method always simulated as a real-time data stream. This was important to ensure that this proposed method did not cause increases in latency that would limit its real-time deployment in our BCI-NFT.

Once the datasets were fed into our detection algorithm, the offline analysis primarily involved adjusting the size of the Fast and Slow MA windows for each participant to maximize detection success. Individualization is important because EEG data patterns differ across individuals. This step is similar to calibration or training datasets collected in other real-time detection methods, although this approach is far simpler and requires minimal pre-training trials.

Finally, although the novel application of this MA method was conceived primarily to address the noise side of the SNR problem, we also aimed to enhance the signal by designing the paradigm to increase engagement in and attention to the movement task. Specifically, we implemented a random GO/NO GO aspect requiring participants to attend to on each trial. This change in the paradigm had a notable effect on neural dynamics, which can be enhanced by increasing task difficulty, complexity or uncertainty (as done here), and movement speed or level of force exerted [40,41] with a resultant downstream effect on detection accuracy.

## 2. Materials and Methods

### 2.1. Participants

We recruited four young adult participants for initial testing of our novel detection method, two with cerebral palsy or CP (CP1 was a 28.9 year old female with bilateral CP, Gross Motor Function Classification System (GMFCS) Level III, which indicates that she is able to walk, although with difficulty and requiring assistive devices; CP2 was a 22.3 year old male with bilateral CP, GMFCS Level II, which indicates that he can walk without assistive devices but has difficulty with stairs and uneven surfaces, and two without neurological injuries designated as neurotypical (NT), (NT1 was a 22.2 year old male; NT2 was a 24.1 year old female).

All participants provided informed consent for our protocol, which was approved by the National Institutes of Health Institutional Review Board (protocol #13-CC-0110).

### 2.2. Description of Our BCI-NFT System

Several modifications to our previously deployed BCI-NFT system [37] were implemented for the current study. First, the ‘preparing to move’ cue, initially designed as a moving horizontal bar, was eliminated because it often caused a negative deflection to occur prior to the cue to move, and the horizontal movement of the bar also caused excessive unwanted head movement, which increased EEG noise. Instead, we instituted a ‘rest’ cue with a countdown displayed in the center of the screen followed by a 2 s preparatory movement phase during which a static circle would fill indicating the timing of a pending cue, Next, a 3 s display of either: a green circle (‘GO’) or a red circle (‘NO GO’) was shown to indicate whether the participant should or should not execute the ankle movement. The new addition here, of a randomly occurring ‘NO GO’ cue in approximately 25% of the trials, was implemented to increase task uncertainty, thereby enhancing participant attention to the task.

**Figure 1:**
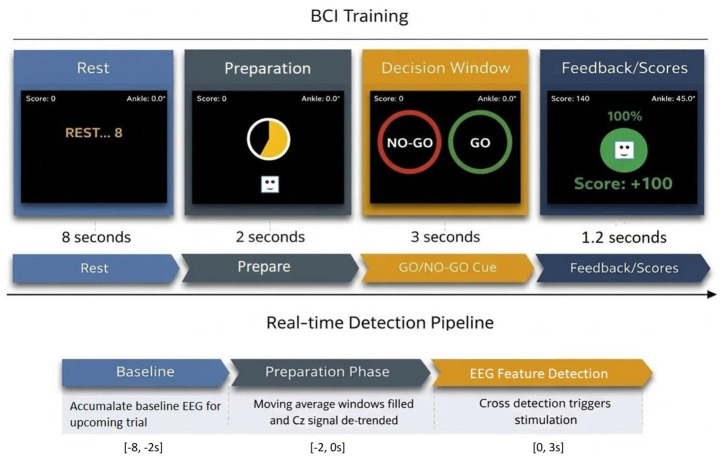
The top of the figure shows the information displayed to a participant throughout a trial and the timing of each phase. The bottom figure shows the simulated real-time pipeline for detection of the crosses between the Fast- and Slow-Moving Averages (MAs) that would subsequently lead to activation of the neuromuscular electrical stimulation unit during active dorsiflexion attempts.

The top row of Figure 1 shows the series of different windows shown on a computer screen to the participant during a trial. Our BCI-NF system is designed as an interactive game where the participant, when cued by the ‘GO’ command, must dorsiflex the ankle as far and as fast as they can with feedback of their movement represented on the screen by a ball which moves upwards into a green circle in the center of the screen. After the ball enters the circle, its background fills proportional to the amount of active dorsiflexion the participant achieved relative to his/her individual target. The target is set individually to challenge each to perform near the limits of their capabilities and, during training, would be progressed as they improve to maintain a high level of engagement. Alternatively, if the participant moves the ankle after a ‘NO GO’ cue, the feedback window displays the words ‘Don’t move’. The feedback screen lasts for 1.2 s, and then another trial begins.

The BCI-NFT training goal is to increase active ankle dorsiflexion’s range of motion and/or angular velocity, with the functional goal of improving foot clearance and gait speed during walking. Since the aim of the current study was evaluation of a new detection algorithm and not motor training, the data collection sessions were identical to the training paradigm except that the neuromuscular electrical stimulation (NMES) was not attached to the participant during the experiment but integrated into the real-time loop for data collection and later offline analysis purposes only. During the offline simulation, when the desired change in trend was identified, our BCI-NFT system triggered electrical stimulation from the LG-8TM Elite Electrical Muscle Stimulator (LG Med Supply, Cherry Hill, NJ, USA) to demonstrate its integration with all other system components.

When this system is utilized for motor training, the pre-movement EEG signal is detected during the GO trials only (the NMES stimulator is inactivated in the NO GO trials) and low-level surface NMES to the tibialis anterior will be activated with the level of NMES determined based on participant feedback at the beginning of the session and held constant throughout the session. The intent is that this should be kept at a comfortable level so as not to discourage the participant from wanting to move. Although the stimulation may assist movement, the main objective is to enhance sensory feedback to the cortex associated with dorsiflexion movement.

### 2.3. Experimental Procedures

During the experiment, participants wore a dry 19-electrode EEG headset (DSI-24, Wearable Sensing, San Diego, CA, USA). The electrodes are spring-loaded and positioned around the headset to offer full head coverage. On average, the DSI-24 headset takes less than 3 min to set up, providing a significant benefit by maximizing patient time for the experiment compared to a wet-electrode EEG system used previously in our BCI-NFT studies (Behboodi et al., 2024 [37]). Once the headset was comfortable and accurately positioned, the real-time impedance monitor in Wearable Sensing’s DSI-Streamer was used to ensure adequate sensor contact on the scalp for each EEG electrode. The DSI-24 headset was directly connected to a central control PC to ensure consistent, reliable streaming (300 Hz) of data from the Cz electrode, with Pz as the online reference into our BCI-NFT system using Wearable Sensing’s lab streaming layer GUI (https://github.com/labstreaminglayer/App-WearableSensing (accessed on 24 January 2025)) to synchronize these data with our MATLAB R2025b detection algorithm. Another difference here compared to our earlier BCI-NFT training paradigm is that we did not apply a filter to only capture the slow frequency potentials (0.5–5 Hz) for detection, thus retaining EEG data in other frequency bands, including alpha (8–13 Hz) and low beta (13–20 Hz).

A microcontroller (Teensy 3.2 (SparkFun Electronics, Boulder, CO, USA)) was connected to the computer which, during an actual training session, would run the MATLAB real-time detection algorithm. However, instead of real-time detection, in this study, the multiple movement trials collected were used to later simulate real-time single trial detection offline.

To record ankle angle and velocity during the trials, two 9-axis inertial measurement units or IMUs (LP-Research, Inc. (Tokyo, Japan) Motion Sensor Bluetooth version 2) were placed on the participant’s lateral shank and dorsum of the foot. The LP-Research sensor fusion algorithms output sensor position and orientation via Bluetooth to a custom Unity application that calculates ankle angle using Euler angles. The exact placement of the sensors varied per participant but was optimized to avoid gimbal lock during dorsiflexion. The BCI-NFT visual interface, which provided instructions and real-time feedback to the participant, was implemented in Python 3.12. The LabRecorder GUI (https://github.com/labstreaminglayer/App-LabRecorder (accessed on 24 January 2025)) was used to communicate between, synchronize, collect, and save all data on a central PC running the BCI, detection, and IMU applications simultaneously.

**Figure 2:**
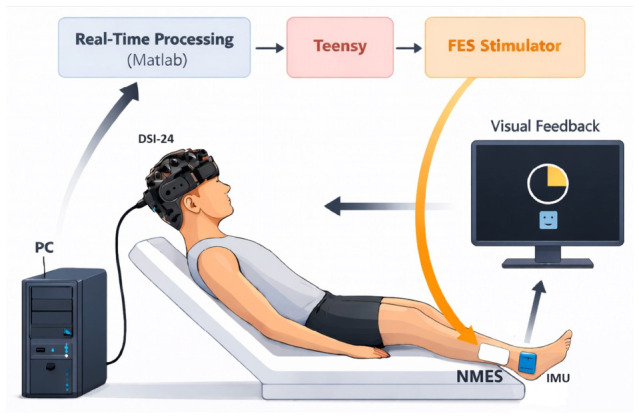
Schematic of our modified Brain–Computer Interface Neurofeedback Training (BCI-NFT) system used to detect EEG features related to motor intent and deliver assistive sensory feedback (neuromuscular electrical stimulation of the tibialis anterior muscle) during ankle dorsiflexion training. EEG signals are acquired using a DSI-24 headset and streamed to a PC for real-time detection. The processed detection signal is then sent to a Teensy microcontroller to trigger stimulation. Two Inertial Measurement Units (IMUs) placed on the participant’s foot were used to track real-time ankle angle and a monitor was used to provide instructions and real-time feedback during training.

Figure 2 illustrates the experimental protocol and its components. Participants sat on an adjustable examination table in a semi-recumbent position, with their knees resting on a small pillow for comfort and their foot extending over the edge of the table to allow the ankle to move freely.

For this study, participants attended a single session with the aim of collecting a minimum of 15 trials (i.e., 15 dorsiflexion movements), with additional trials attempted if the participant was willing and ankle performance had not deteriorated due to fatigue. No calibration trials were performed; however, we did allow each participant to become familiar with the task and the BCI-NFT system so that they could respond to the best of their ability during data collection. During a trial, the BCI-NFT system was activated whereby the participant was first shown an 8 s countdown displayed on the screen with the word ‘Rest’, then the 2 s preparation phase indicated by the filling yellow circle, followed by a 3 s ‘GO’ or ‘NO GO’ cue, with feedback provided for 1.2 s. In a GO trial, the feedback provided on the screen is directly controlled by the participant’s real-time ankle dorsiflexion angle as recorded by the IMUs. Trials were collected in blocks, and each block concluded after 5 GO trials had been collected.

During the experiment, each participant was given practice trials with the system to make sure they were able to perform the task reliably and felt comfortable with all procedures. We also encouraged participants to notify us if they wanted to take a break or were experiencing any discomfort.

After minimal practice, all were able to perform the task well, except for CP1 who was initiating movement too soon, likely starting in the preparation phase. For this participant, we decided to remove the preparation cue and left the screen blank for 2 s before the GO/NO GO cue, which seemed to resolve the issue. This participant also needed more time to move, which caused us to shift the detection window 0.25 s later, and also requested more rest between trials. CP2 experienced fatigue and reported some mild ankle discomfort from the repeated movements, so we stopped the session at that time. NT2 reported mild discomfort from the cap which exerts slight pressure on the scalp for better electrode connectivity, so we stopped the session at that point.

### 2.4. Simulation of Real-Time Detection of Pre-Movement EEG Changes

We chose to leverage a time-series forecasting technique that applies a pair of MA windows to time-series data to detect changes in the trend, or underlying direction, of the signal. These trends are predicted when the shorter-term Fast MA crosses either under or over (i.e., becomes lower than or surpasses, respectively) the longer-term Slow MA that may represent the late component of the MRCP or onset of ERD within a window of −400 ms to +100 ms of ankle movement onset.

A MATLAB-based script was used to load the synchronized time series data for Cz EEG data, ankle position, and event labels (GO/NO GO), into a pipeline to assess the feasibility and accuracy of using MAs to detect trends while still simulating real-time deployment. First, all GO events were identified, and the Cz data for these were epoched from [−6 s, 5 s] relative to the start of the preparation phase. The last 6 s of each 8 s rest period were used for the baseline. A linear model was fit to each baseline period to detrend remaining samples in each trial. The optimized MA windows, Fast and Slow, were enabled at the beginning of the preparation period. Both windows are causal, using only current and previous samples, so, once filled, do not incur any latency. As shown on the bottom half of Figure 1, at the conclusion of the 2 s preparation period, the detection window opens for 3 s, or the latencies of [2 s, 5 s] from the onset of the preparation period.

**Figure 3:**
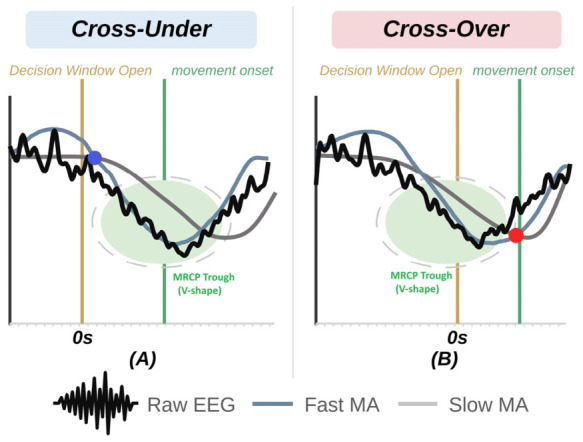
This illustration shows the Fast- and Slow-Moving Averages (MAs) based on detrended EEG data shortly before and after the detection window opens, as indicated by a yellow line, and is: (**A**) identifying the steep slope of a MRCP during a cross-under indicated by a Blue marker (Fast MA crosses under the Slow MA) or (**B**) the reversal in direction after the negative peak in the MRCP indicated here by a cross-over indicated by a Red marker (Fast MA crosses over the Slow MA). Both are shown here as occurring before the movement onset, indicated by a green line.

When the detection window opens, the stimulator becomes active awaiting a trigger from a detection, identified as a crossing of the Fast and Slow MAs (Figure 3). The detection could be caused by one of two occurrences: (1) a cross-under—if the Fast MA crosses under the Slow MA (depicted by a Blue marker), this confirms a local maximum and predicts a downtrend, or (2) a cross-over—if the Fast MA crosses above the Slow MA (depicted by a Red marker), this confirms a local minimum and predicts an upward trend. The algorithm could be set to only detect Blue or Red crosses, or a hybrid (Gold) method could be set to choose the first cross, regardless of whether it is a cross-under or cross-over, as the detection point. This decision fluctuates based on the relative positions of the Fast and Slow MA lines when the detection window opens and enables the type of cross to vary by trial.

In the offline analysis for each participant, different combinations of window sizes for the Fast MA and Slow MA were evaluated to optimize detection accuracy (i.e., occurrence of crosses within the target time period from −400 ms to +100 ms relative to ankle movement-onset as detected by the IMUs). Combinations of Fast MA window sizes of 50, 100, and 150 samples, with larger Slow MA window sizes of 100, 150, and 200 samples, were tested. Detection accuracy was computed for each configuration, with the highest yielding combination used for the subsequent analysis of the best detection method for each participant (Blue only, Red Only, or Gold [Blue or Red depending on which occurs first]). The same data here were used for determining the optimal MA window size and for determining the best detection method. In future studies, a small sample of data can be collected at the start of each session before the training trials to optimize MAs window sizes prior to real-time MRCP or ERD detection.

### 2.5. Outcomes

The occurrence of detections in all GO trials in the 500 ms window pre-movement (−400 ms) to immediately post-movement onset (+100 ms), which we term the target window, (number and % of trials and whether they were over or under crosses) and their timing (in ms) with respect to movement onset were recorded for each participant. The percentage of GO trials with a detection within the target window was computed as the true positive rate (TPR). If a detection occurred but was outside the target window, it was recorded as a false positive, and if no detection occurred, it was a false negative.

We also performed a more detailed analysis of each of the MA crosses that indicated a detection by evaluating the data in each epoch first, including all frequencies as in the real-time simulation, and then only including the slow frequencies (0.5–5 Hz). For each detection, we evaluated whether the cross had identified the steep downward slope or the subsequent upward deflection characteristic of the MRCP and/or the start of an ERD. An independent review of each trial to determine whether the detection successfully identified a MRCP and/or ERD was conducted by two investigators, each with more than a decade of EEG experience, with any differences resolved through discussion. The aims of this analysis were to determine the extent to which our algorithm detected a physiological event and to compare results from the slow frequency data alone, which we had used previously, to the larger frequency range used here.

## 3. Results

Briefly, the datasets collected for each participant were iteratively examined using the three different sizes of the Fast- and Slow-Moving Averages’ windows and their possible combinations, with respect to each of the three detection methods (cross-over, cross-under, or hybrid that detected whichever occurred first after the detection window opened) to identify which solution produced the highest true positive rate of EEG detection for each individual. In an actual training study, this step would need to be completed prior to training, using a small sample of data for each participant to set individualized parameters for real-time detection during training.

Next, we examined each of the detection points in every trial to determine whether these were identifying a true physiological event, i.e., either a MRCP or an ERD, to further validate the use of this algorithm.

### 3.1. Detection Results

**Figure 4:**
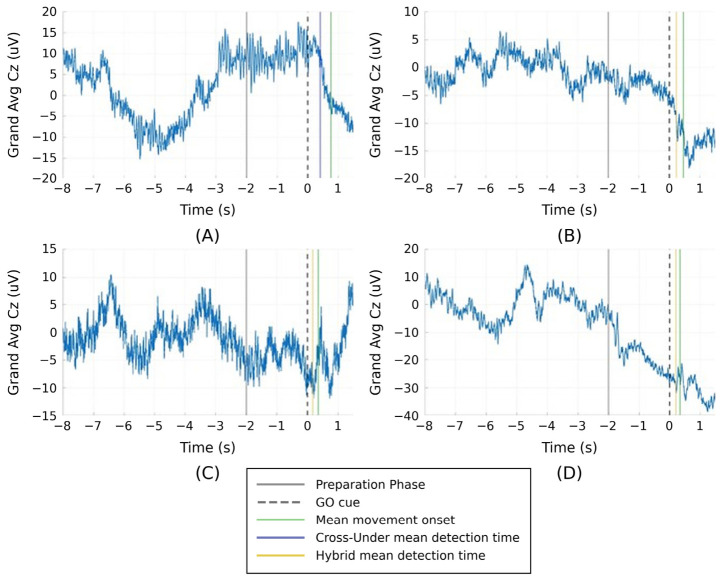
Grand means of Cz data for all trials for each participant: (**A**) CP1, (**B**) CP2, (**C**) NT1, (**D**) NT2. Data include several seconds prior to the GO cue (dotted black line), the mean detection time with method indicated by line color (blue for CP1, and gold for CP2, NT1, and NT2), and mean movement onset (green line).

A summary of the real-time simulation data is shown in Figure 4, depicting the grand mean Cz EEG data across all trials, and the mean timing of all detections (blue or gold line) with respect to the movement onset (green line) for each participant. Mean movement onset and mean detection times are presented in Table 1 for each participant. Notably, all participants initiated movement within 1.5 s after the GO cue (dotted line indicated as time 0), and all mean detections also occurred before movement onset. Detection was active at the beginning of the detection window, which started when the GO cue was presented. An exception was made for CP1 who demonstrated consistently delayed movement reaction times due to her neurological involvement, so the detection window was delayed by 0.25 s after the GO cue to compensate for this and thus avoid detection and stimulation too far from movement onset.

These summary data represent the results from the optimized Fast/Slow MA windows determined for each participant in the initial iterative analysis, which were: 100/150 for CP1, 50/200 for CP2, 100/150 for NT1, and 100/150 for NT2 by evaluating the TPR (percent of all trials for which detection had occurred within the target plasticity window of the −400 ms to +100 ms of movement onset) for each MA size and combination for each of the three methods (Blue, Red, or Gold [hybrid]) for each participant. For CP1, the Blue (cross-under) detection method, which ignored any cross-overs that may have occurred first, was the most accurate. For the other three participants, the hybrid Gold method was the most accurate. This method selected either a Red or Blue detection depending on which occurred first, which was based on the relative position of the Fast and Slow MAs when the detection window opened. Supplementary Figures S1–S4 depict the individual trial data showing each of the detected crosses indicated by the type (Blue, Red, or Gold overlaying either a Red or a Blue) and the timing, with respect to movement onset, for each participant and the detection method chosen.

Table 2 shows TPR data for each participant as well as the FPR or the percentage of all detections outside of the desired time window that are considered less optimal and potentially appropriate for fostering adaptive neuroplasticity, and the number of false negatives (FN) or trials where no detection occurred even though the participant moved. Since only GO trials were evaluated and movement was confirmed to have occurred in each trial, we were not able to compute true negatives. For CP1, the Blue (cross-under) detection method which ignored cross-overs that may have occurred first, was the most accurate (highest TPR). For the other three participants, the Gold method, where whether a Red or Blue detection was selected first varied depending on the relative position of the Fast and Slow MAs when the detection window opened, was the most accurate.

The mean TPR ranged from 73.3% to 94.2% with a mean of 85.9% across all participants. The TPR is easy to verify when participants can execute the desired movement, as was the case in this study. The FPR ranged from 5.9% to 26.7% with a mean of 14.1% across all participants. There was only one false negative in a single participant (trial with no detection).

### 3.2. Trial-by-Trial Analysis of Each Detection to Identify a MRCP or an ERD

What is often difficult to determine, and thus rarely reported in studies on real-time detection accuracy, was whether the method was successful on an individual trial basis of detecting an identifiable physiological signal, specifically here, for the later component of the MRCP or a different pre-movement feature of the EEG such as the ERD. Results from the independent review of each trial by two of the authors are summarized in Table 3. These showed that MRCPs were evident in 80% of the trials for each of the two participants who were neurotypical, with ERD not detected in one, and detected in 20% of trials in the second with only one trial where the ERD alone was detected. Results for the two participants with CP differed from those who were neurotypical as well as from each other. For one participant with CP, MRCPs were detected in 70% of trials, and ERDs were detected in 32% of trials with 3 of 18 ERD detections without a MRCP. For the second participant with CP who had more neurological involvement, MRCPs were seen in only 23% of trials; however, ERD was evident in 87% of trials in this individual. This same participant also had the lowest TPR and highest FPR.

### 3.3. Topographical Maps of Alpha Band Power (8–13 Hz) in Cz During the Experiment

Changes in alpha power across the different phases of the experiment are depicted in Supplementary Figure S5 for each participant. All data are presented as a percentage relative to each participant’s baseline. A blue color indicates desynchronization, which is expected to be greatest just before or after movement onset. Each participant shows ERD as expected around the time of movement onset, but the magnitude of ERD and the time at which it reaches the highest relative magnitude varies across participants.

## 4. Discussion

### 4.1. Summary of Previous Efforts to Improve Real-Time Detection of Pre-Movement EEG Signals

Real-time detection of EEG signals prior to movement (or the imagination of movement) onset remains a significant technical challenge for the field with multiple studies claiming some level of success from various methods, although no one method has demonstrated superiority. Major remaining challenges include: the inherent low signal-to-noise ratio of surface EEG signals, especially on an individual trial basis, computation speed requirements of more sophisticated methods that limit real-time detection, and the time and effort required to train certain models prior to implementation. Some studies claim success from offline analyses with methods that are problematic for real-time single-trial implementation. Success rates in many studies are based solely on whether a detection occurs within a pre-specified time window that varies across studies without clear identification of which feature of the EEG signal is being detected in some instances. MRCPs are a desirable detection target because they are endogenous signals that do not require extensive BCI experience or training [42]. Although the underlying premise in many studies is that the late pre-movement steep negative peak in the MRCP is what is being detected, direct verification of this is rarely presented. Many studies focus on developing a robust classifier to distinguish rest states from movement intention or preparation, often with less emphasis on precise timing of detection with real or imagined movements. Other studies have utilized detection timing-based on pre-training data instead of real-time detection [35], or have supplemented real-time detection with automatic device activation if detection had not yet occurred near the end of the target window in a given trial [37]. According to Hebb’s Law, if two neurons fire within 400 ms of each other, their connectivity will be strengthened [43], so if the intention of BCI-NFT is to foster adaptive neuroplasticity, timing of detection to activate a device, at least this close to movement onset, is critical.

### 4.2. Evaluation of the Success of Our Algorithm Compared to the Literature

In this study, we aimed to improve real-time detection accuracy and timing of the characteristic features of the late MRCP or pre-movement ERD during motor training by utilizing a time series forecasting strategy that detects emerging data trends using two MA windows of different sizes. As with other studies, the detection window was restricted, in this case to 400 ms prior to or coincident (+100 ms) with movement onset, and only the GO movement trials were assessed so we could not report false positives, although this could be expanded in future studies evaluating this method by assessing whether detections occur in NO GO trials when the person does not move.

The mean TPR here was 85.9%, with a mean FPR of 14.1% and a mean timing of 182 ms prior to movement onset. This level of performance is equal or arguably superior to previous applications evaluating similar movement tasks, including our own earlier results. Niazi et al. (2012) achieved a TPR of 67.2 ± 7.9% using multi-channel MRCP detection for real-time motor imagery of ankle dorsiflexion [44], whereas Xu et al. (2014) reported a TPR of 79% ± 11% at a detection time of 315 ± 165 ms from movement onset using a real-time, multi-channel system during ankle dorsiflexion [45]. In a clinical study, Mrachacz-Kersting et al. (2018) reported a TPR of 71 ± 3% for their online associative BCI intervention [46]. In a previous study using the same basic BCI-NFT system with a hybrid real-time transfer learning based single-channel detection that activated electrical stimulation (or alternatively, stimulation was activated at the end of the target window if no detection occurred) Behboodi et al. (2024) [37], reported 80.8% detection accuracy for the same ankle dorsiflexion task in a similar population as the current study (eight neurotypical adults and one child with CP) [37]. A recent study by Dong and coauthors [47] who proposed a real-time method to better identify the MRCP for improved timing of exoskeleton control using convolutional neural networks (CNN) and transfer learning, reported a TPR of 82.7% with a timing of −483.9 ms. While their accuracy approached ours, our timing results were far closer to those deemed critical for optimizing adaptive neuroplasticity during training. The other important difference in our algorithm was that it was capable of identifying both MRCPs and ERD, the combination of which has been shown to produce more accurate results as stated in the introduction [27].

While studies may continue to aim for even higher accuracy, it is possible that currently reported accuracy ranges may represent the realistic likelihood of identifying MRCPs on a trial-by-trial basis in real time given their variability in amplitude and timing within and across participants and tasks. Our examination of individual trials found that our detection method picked up MRCPs in 80% of trials in the two participants who were neurotypical. In the participants with CP, MRCPs were identified at the time of the detection in 70% of trials for one and 23% in the other, who interestingly showed a far higher rate of ERD detection (87%) than any of the others. Although ERDs had a minimal, if any effect, on the TPR in the other three participants, our data suggest that ERD detection could be an alternative detection strategy in those cases when MRCP detection is not as successful.

Bai and colleagues [26] proposed that the true positive rate was the most important metric for evaluating clinical success in fostering adaptive neuroplasticity. Additionally, they made the intriguing suggestion that accurate detection of unwanted movements could lead to innovative therapeutic strategies to prevent or correct these before they occur. For BCI-NFT training, timing of detection to activate a device very close to movement onset is also a critical element for fostering neuroplasticity. The mean timing of detection here was well within this window. Others have suggested that the amplitude of the MRCP may be useful as a valid indicator of increased neural efficiency as a result of motor training and even for tracking neural recovery in stroke, especially given the close association between EEG and motor changes [40,48]. The benefits of enhanced timing and accuracy for controlling brain–computer interfaces and for reducing or eliminating the need for extensive training before achieving the ability to control an external device are of great importance in the field, so we contend that our proposed method clearly warrants serious consideration for this application as well.

### 4.3. State-of-the-Science in BCI-Based Neurofeedback Training for Neurological Disorders

The primary focus of our research is on evaluating and advancing the effectiveness of BCI-NFT motor training for individuals with CP and other neurological disorders. We recognize that while the results here are preliminary and were directed solely at enhancing real-time detection accuracy, the clinical effectiveness of this detection method in combination with our existing paradigm needs to be demonstrated in a motor training study in a patient population. A recent review on the state-of-the-science in NFT by LeFranc et al. [49] highlighted its current overall effectiveness as an adjunctive therapy in stroke, despite the many technological challenges that remain. The effectiveness of NFT has not yet been adequately demonstrated in individuals with CP. These authors [38] emphasized the continued need for engineering development in the detection and utilization of brain signals to achieve significant advances in neuroscience and neurorehabilitation. They touted EEG as the most highly utilized technology for NFT given its ease of use and tolerability, with continuing improvements in hardware and software (e.g., dry electrodes, more targeted detection regions, advances in signal processing, advent of hybrid approaches, incorporation of more relevant feedback, etc.) making this even more effective and user-friendly for clinical applications. Interestingly, these authors focused primarily on studies that involved self-modulation of cortical rhythms in the mu and beta bands as the EEG control signal which requires significant training, rather than detecting MRCP features. In contrast, a more recent review aiming to comprehensively summarize all literature related to brain–computer interfaces, Khorev and colleagues [50] had a more expansive view on real-time detection of brain signals for device control. Interestingly, in a network diagram they produced on the universe of BCI literature, the strongest nodes that emerged were EEG, stroke, rehabilitation and motor imagery, demonstrating the high level of research interest and effort directed towards these applications in the BCI field. This review also noted that brain signal detection has large clinical as well as societal implications in being able to detect and perhaps even affect the control of brain activity, with achieving precise timing between thoughts and action being a critical component of their success.

### 4.4. Strengths and Limitations

The strengths of this study are the innovative and easily implemented methodology, and the associated positive results in terms of accuracy and timing, as well as verification of the physiological events identified by the detection method. Our MA approach requires the specification of two key parameters for each participant: the duration of the fast and slow windows with three options for each selected here, resulting in nine possible combinations. Each combination was then evaluated with respect to three different detection strategies. Although the goal of this paper was to establish the initial feasibility of our novel MA approach, future analyses with additional participants and trials will enable a formal sensitivity analysis to examine the effect of these model parameters on success rate, timing, and false positive and negative rates. Importantly, the results in this study were obtained with modifications to our earlier approach that confer critical experimental and methodological advances, including the use of a single channel (Cz) of data collected from a dry EEG headset with no dedicated artifact rejection methods, thus indicating the potential for efficient clinical deployment.

The major weakness was that real time was simulated and optimized prior to analysis. For true real-time implementation, fine tuning of the MA windows, the detection method, and detection window timing would be necessary, which would involve collecting a small amount of data to optimize detection prior to a training session. Personalization of detection methods and features detected may be particularly necessary in those with neurological disorders.

The small sample is also a limitation because it is inadequate to demonstrate the full range of responses to the detection algorithm that could potentially occur or for statistical generalization of our findings at the population level. However, the aims of this study were to present a novel proof-of-concept method for detecting EEG activation prior to or simultaneous with movement onset, and to assess the feasibility of our detection method in an initial cohort of four participants: two neurotypical individuals and two individuals with cerebral palsy. Our detection method was shown to be feasible and accurate, providing preliminary evidence that our approach might generalize across neurotypical and neurologically injured populations. These findings present a compelling case to investigate this detection approach in a larger, controlled clinical trial, or in other BCI applications.

## 5. Conclusions

The primary objective of this study was to evaluate a novel detection method commonly used in time-series forecasting, which identifies crosses between fast (short) and slow (long) moving average windows to detect negative deflections in slow movement-related cortical potentials (MRCPs) or event-related desynchronization (ERD). This approach was developed to enable real-time, training-free detection of motor activity from scalp EEG prior to or at movement onset to trigger functional electrical stimulation (FES) during ankle dorsiflexion training. The results from our feasibility study showed a true positive rate of 85%, with detection occurring on average 182 ms prior to movement across four participants, retaining critical timing for Hebbian-based learning through neuroplasticity from both voluntarily and FES-activated muscle. These results were consistent across both neurotypical participants and those with cerebral palsy, with post hoc analyses demonstrating that our detection method captured well-known correlates of movement-related cortical activity. All results were achieved with a dry electrode headset (DSI-24) using a single channel (Cz) for simulated real-time EEG detection, enabling efficient translation to clinical applications. Collectively, our results provide the impetus for a larger clinical trial to confirm our detection method’s robustness across a wider range of individuals with CP with differing degrees of motor involvement, and to evaluate the therapeutic effect of the current BCI-NFT system to improve ankle dorsiflexion and thereby gait function. Implementation of this approach for other BCI applications also warrants exploration.

## Figures and Tables

**Table 1:** Mean movement onset, mean detection times and standard deviations across all trials for each participant from the start of the GO cue, designated as Time 0.

	CP1	CP2	NT1	NT2
Mean Movement Onset (s)	0.76 ± 0.16	0.46 ± 0.09	0.35 ± 0.05	0.34 ± 0.05
Mean Detection Time (s)	0.49 ± 0.09	0.24 ± 0.17	0.23 ± 0.13	0.22 ± 0.12

**Table 2:** True positive rates (TPR), false positive rates (FPR), and the number of trials with no detections or false negatives (FN) computed using the detection method deemed the most effective for each participant.

	CP1	CP2	NT1	NT2
Detection Method	Blue	Gold	Gold	Gold
True Positive Rate (TPR) %	73.3	94.2	90.0	86.1
False Positive Rate (FPR) %	26.7	5.7	10.0	13.9
False Negatives (FN) #	0	0	0	1

# represents number of occurrences.

**Table 3:** Mean results of detections (Moving Average or MA crosses) for each participant and detection strategy (cross-over [Red], cross-under [Blue], or hybrid [Gold]), including number and percentage of detections of a slow movement-related cortical potentials (MRCP) and/or an event-related desynchronization (ERD), and number of trials with only an ERD.

Participant (Number of Trials/Number by Method)	MA Cross Type	MRCP (%)	ERD (%)	ERD NO MRCP
CP1 (15; 15 Blue)	Blue	8 (23)	13 (87)	5
	Red	0 (0)	0 (0)	0
	**TOTAL**	**8 (23)**	**13 (87)**	**5**
CP2 (43; Gold; 25 Blue, 18 Red)	Blue	20 (47)	13 (30)	1
	Red	10 (23)	5 (12)	2
	**TOTAL**	**30 (70)**	**18 (32)**	**3**
NT1 (20; Gold; 6 Blue, 14 Red)	Blue	3 (15)	0 (0)	0
	Red	13 (65)	2 (10)	0
	**TOTAL**	**16 (80)**	**2 (0)**	**0**
NT2 (35; Gold; 10 Blue, 24 Red, 1 No detection)	Blue	10 (29)	2 (6)	0
	Red	18 (51)	5 (14)	1
	**TOTAL**	**28 (80)**	**7 (20)**	**1**

## Data Availability

The protocol under which these data were collected is still ongoing and will not be available until the project is completed.

## Supplementary Materials

The following supporting information can be downloaded at: https://www.mdpi.com/article/10.3390/bioengineering13050561/s1, Figures S1–S5: Figure S1 shows the individual trial data for participant CP1 from the start of the GO cue. The target detection window (−400 ms to +100 ms) is shown by the shaded area and it determined relative to the movement onset shown by a green line which varies slightly from trial to trial. the detection point is indicate by a blue marker, or a red or blue marker encircled by gold depending on the detection method used and whether the detection indicated a Cross-under (blue) or Cross-over (red). Figure S2 shows the individual trial data for participant CP2 from the start of the GO cue. The target detection window (−400 ms to +100 ms) is shown by the shaded area and it determined relative to the movement onset shown by a green line which varies slightly from trial to trial. the detection point is indicate by a blue marker, or a red or blue marker encircled by gold depending on the detection method used and whether the detection indicated a Cross-under (blue) or Cross-over (red). Figure S3 shows the individual trial data for participant NT1 from the start of the GO cue. The target detection window (−400 ms to +100 ms) is shown by the shaded area and it determined relative to the movement onset shown by a green line which varies slightly from trial to trial. the detection point is indicate by a blue marker, or a red or blue marker encircled by gold depending on the detection method used and whether the detection indicated a Cross-under (blue) or Cross-over (red). Figure S4 shows the individual trial data for participant NT2 from the start of the GO cue. The target detection window (−400 ms to +100 ms) is shown by the shaded area and it determined relative to the movement onset shown by a green line which varies slightly from trial to trial. the detection point is indicate by a blue marker, or a red or blue marker encircled by gold depending on the detection method used and whether the detection indicated a Cross-under (blue) or Cross-over (red).
