# Supplementary material for: Novel Time-Series Forecasting Method to Enhance Accuracy of Real-Time EEG Detection for BCI-Based Neurofeedback Motor Training in Individuals with Cerebral Palsy and Other Neurological Disorders
Source: Bioengineering (Basel). 2026 May 16;13(5):561. doi: 10.3390/bioengineering13050561 (PMC13203816; doi:10.3390/bioengineering13050561)
Supplement: Supplementary file 1 [file bioengineering-13-00561-s001.zip › bioengineering-4247745-supplementary.pdf]

bioengineering

MDPI

Supplementary Figures

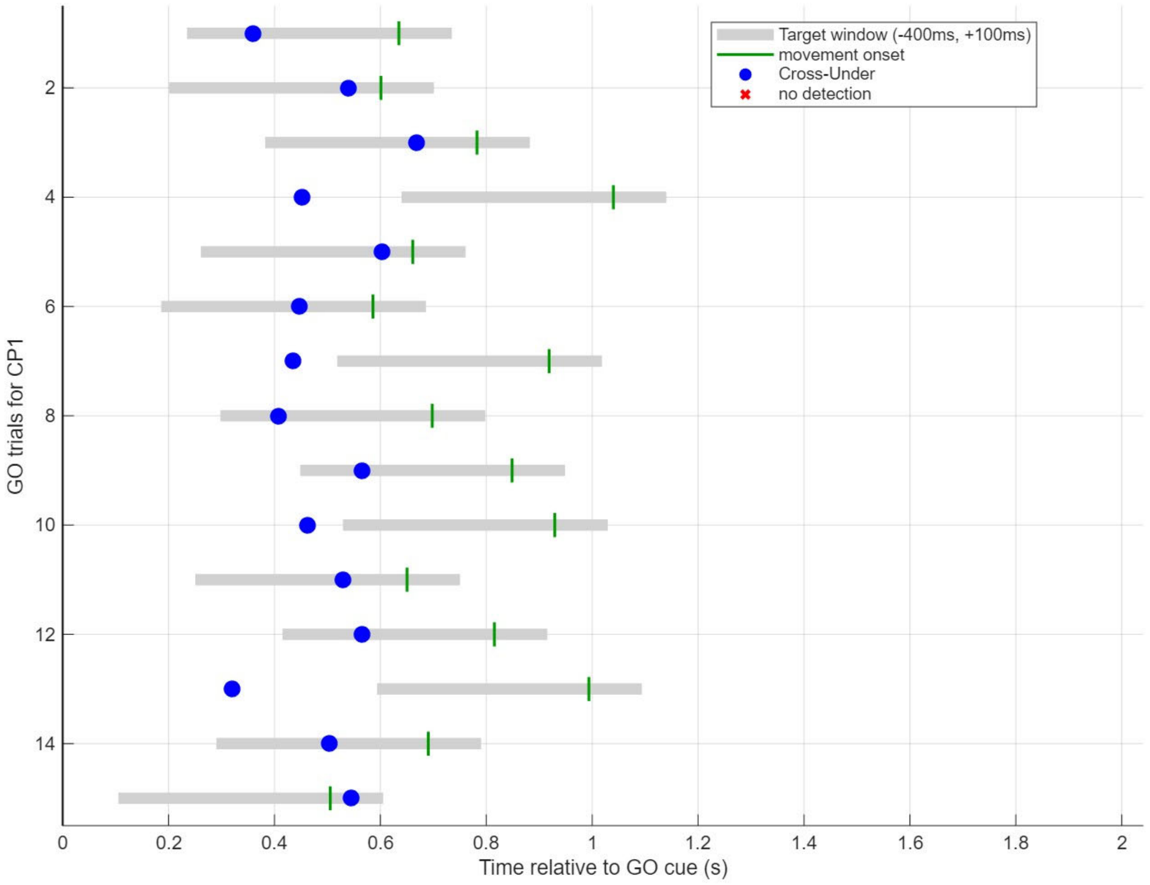

Figure S1

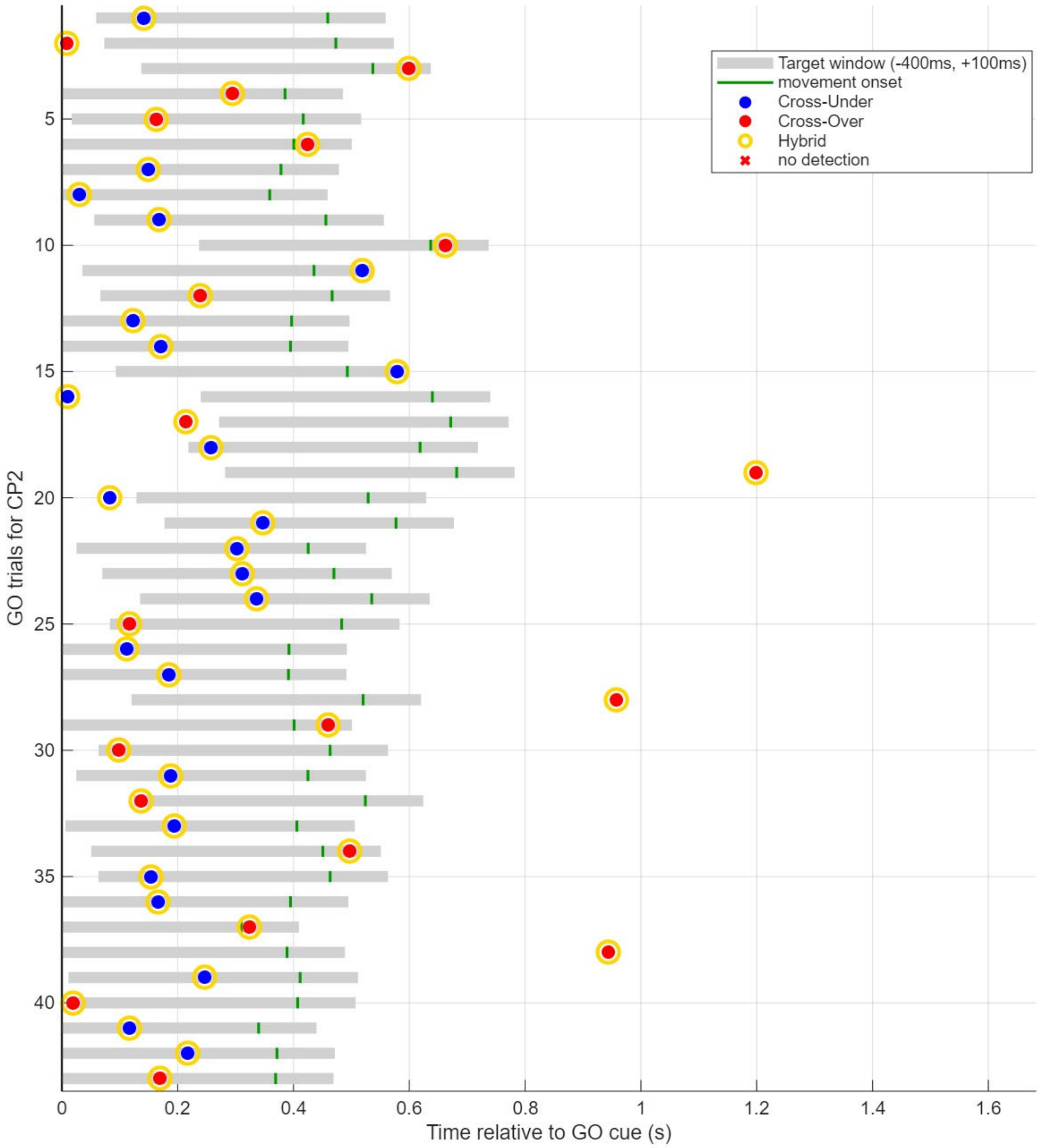
Target window (-400ms, +100ms)
movement onset
Cross-Under
Cross-Over
Hybrid
no detection
GO trials for CP2
5
10
15
20
25
30
35
40
0
0.2
0.4
0.6
0.8
1
1.2
1.4
1.6
Time relative to GO cue (s)

Figure S2

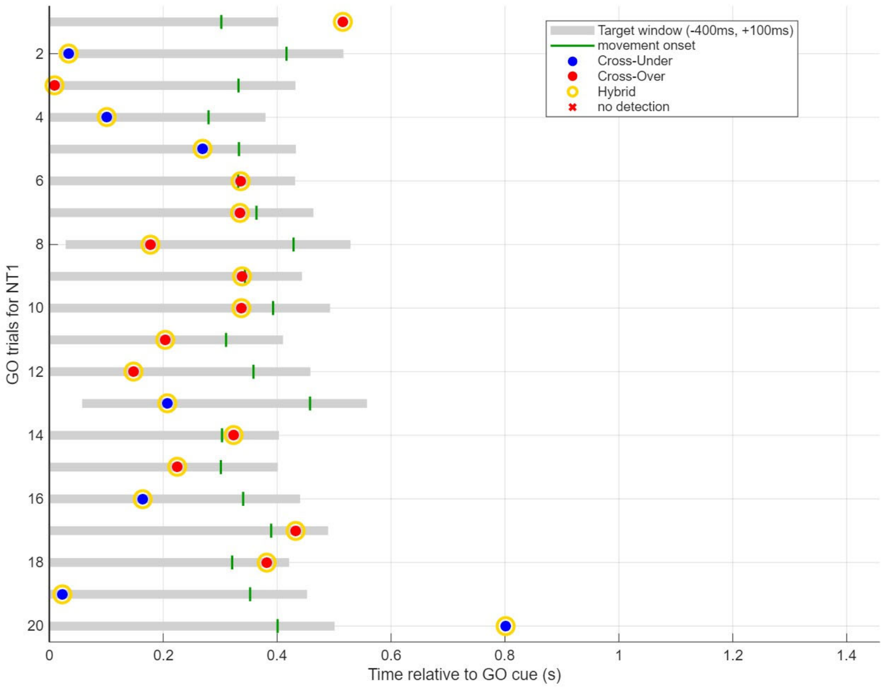
Target window (-400ms, +100ms)
movement onset
Cross-Under
Cross-Over
Hybrid
no detection
GO trials for NT1
2
4
6
8
10
12
14
16
18
20
0
0.2
0.4
0.6
0.8
1
1.2
1.4
Time relative to GO cue (s)

Figure S3

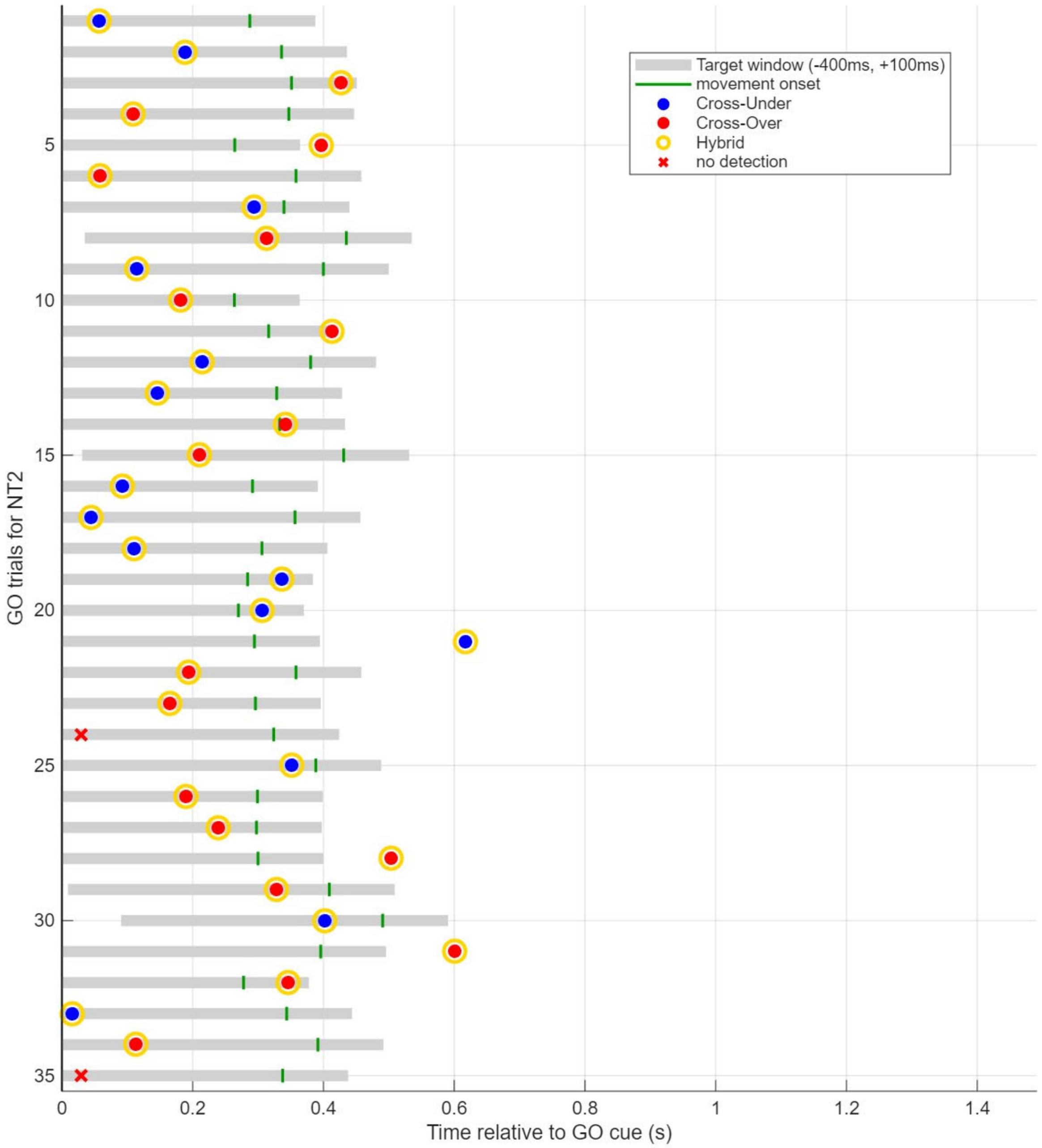

Figure S4

Figures S1-S4 show the individual trial data for each participant (CP1, CP2, NT1, NT2, respectively) from the start of the GO cue. The target detection window (-400ms to +100ms) is shown by the shaded area and it determined relative to the movement onset shown by a green line which varies slightly from trial to trial. the detection point is indicate by a blue marker, or a red or blue marker encircled by gold depending on the detection method used for each participant and whether the detection indicated a Cross-under (blue) or Cross-over (red).

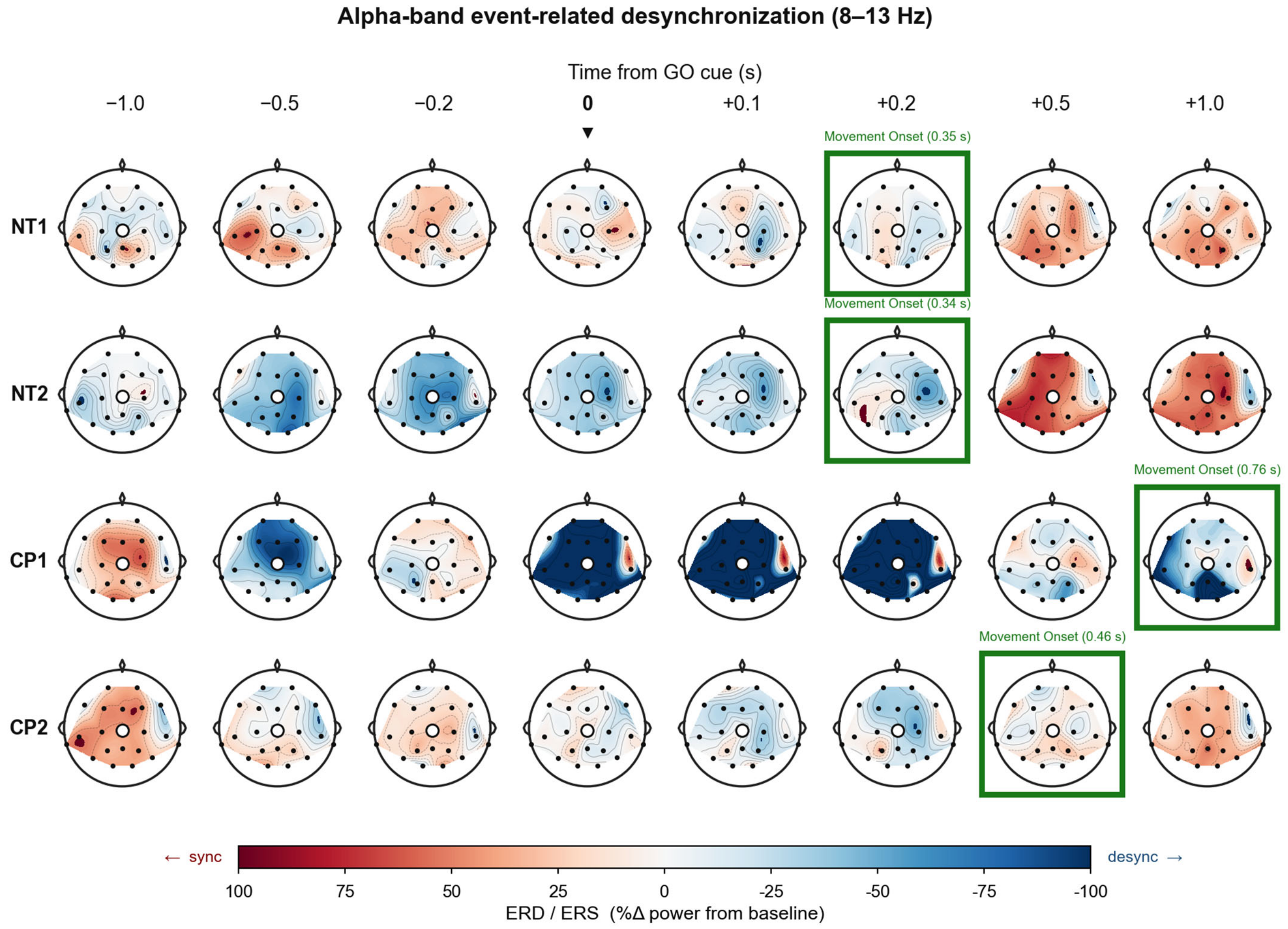

Figure S5

Figure S5 shows the alpha band event related changes in power during the course of the GO trials for each participant. Scalp topographies of alpha band power (8-13 Hz) are relative to the pre-Cue baseline in two neurotypical participants (NT1, NT2) and two participants with cerebral palsy (CP1, CP2). Each brain map represents the average event-related desynchronization/synchronization (ERD/ERS) at a selected window in time from the GO cue. ERD/ERS is expressed as a percent change in alpha power from the 6s pre-cue baseline. EEG data were collected with the DSI-24 cap (21 scalp electrodes at 300 Hz) and bandpassed filtered at 8-13 Hz. The white circle represents the Cz electrode. Green boxes highlight the time window for which movement onset occurred on average for each participant.
